# Nano-casted N-Doped Carbon Created From a Task-Specific Protic Salt and Controlled Porous Glass

**DOI:** 10.3389/fchem.2019.00767

**Published:** 2019-11-27

**Authors:** Varun Singh, Mikhail Gantman, Thangaraj Selvam, Maximilian Münzer, Dirk Enke, Wilhelm Schwieger

**Affiliations:** ^1^Department of Chemical and Biological Engineering, Institute of Chemical Reaction Engineering, Friedrich-Alexander University of Erlangen-Nuremberg (FAU), Erlangen, Germany; ^2^Faculty of Chemistry and Mineralogy, Institute of Chemical Technology, Universität Leipzig, Leipzig, Germany

**Keywords:** N-doped carbon, macroporous carbon replica, direct carbonization, controlled porous glass, nano-casting

## Abstract

3-dimensionally interconnected macroporous carbons are versatile materials that can be used in catalysis, electrochemical devices, and separation technology. Herein, the synthesis of a nitrogen doped carbonaceous material with a well-defined nanoarchitecture via nano-casting is demonstrated. A novel carbon source, a task-specific protic salt, has been proposed to create nitrogen doped carbon by direct carbonization within the pores of controlled macroporous glass. After the removal of macroporous glass from the composite using an aqueous sodium hydroxide solution and upon further heat treatment, an oxidation resistant doped carbon with high nitrogen content (6 mass %) is obtained. The materials formed during the different stages of the nano-casting process exhibit interesting properties such as hierarchical porosity, very high nitrogen content (15 mass %), and increased oxidational stability. A combination of different properties to create tailor-made materials for different applications using this technique is possible.

## Introduction

Protic salts (PS) are an emerging class of materials for creating doped carbon because they can be directly carbonized by a simple heat treatment step (Zhang et al., 2015b). Moreover, they offer the distinct advantage of control over the chemical character of the prepared carbon by simple tuning of the chemistry of the constituents of the PS. Synthesis of PS is straightforward as it involves neutralizing an organic base with a mineral acid. Previous reports on direct carbonization of PS were focused on micro- (pore size < 2 nm)/mesoporous (pore size between 2 and 50 nm) doped carbons prepared with or without an inorganic porous scaffold. These studies show that by swapping the chemical constituents of the PS, micro- and mesoporosity can be controlled (Zhang et al., 2014a). A precise control of mesoporosity can be achieved by applying an inorganic mesoporous scaffold with the well-known technique of nano-casting (Paraknowitsch et al., 2010b; Fellinger et al., 2012; Hasché et al., 2012; Sakaushi et al., 2015; Zhang et al., 2015a; Tzialla et al., 2016).

**Figure 1:**
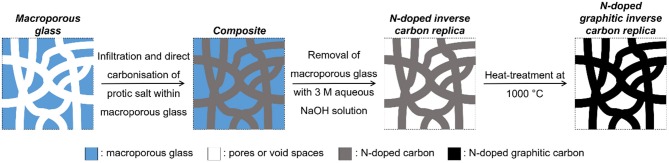
Schematic of the nano-casting process as adapted for this study.

This study applied nano-casting for creating well-defined macroporous doped carbons. Nano-casting for creating porous carbon principally involves: (i) filling the pores of an inorganic porous scaffold with a carbon precursor, (ii) carbonizing the composite, and (iii) removal of the inorganic porous scaffold (Xia et al., 2010) (schematic as applied in this study displayed in Figure 1). Nano-casting offers a high level of flexibility in fabricating well-defined porous architecture and has the following advantages:

- By choosing an appropriate carbon precursor, easy graphitization at relatively low temperatures can be achieved (Parmentier et al., 2004) and highly specific dopants can be incorporated (Paraknowitsch et al., 2010a; Fechler et al., 2013; Zhang et al., 2014a).
- By selection of an inorganic scaffold, nano-casting can provide flexible control over a wide range of pore sizes ranging from micropores to macropores that other, simpler synthesis routes like “soft-templating” cannot (Xia et al., 2010). Moreover, for most catalytic applications, the distinct advantage of periodicity that soft-templating can offer is superfluous (Rolison, 2003). Finally, the cheapest route for producing porous carbon through pyrolysis of biomass-based or petro-based feedstock is unable to rationally control the pore size distribution (White, 2015). Based on the above reasoning, amorphous, inorganic scaffold such as porous glass that have well-defined spatial dimensions and are commercially available can be readily used to full-advantage in nano-casting.
- It has been reported that varying the dilution of carbon-source during the pore infiltration of the inorganic scaffold can have a significant effect on the pore size distribution of the carbon finally derived from nano-casting (Lu et al., 2004). Thus, the step of pore-infiltration grants another degree of freedom to control the modal distribution of porosity of the prepared carbon.
- Depending on the temperature used during the carbonization, the dopant concentration (Paraknowitsch et al., 2010a; Eisenberg et al., 2016) and the degree of graphitization can be tuned (Vergunst et al., 2002).

The process of nano-casting leads to a so-called “inverse replica” structure wherein the pores of the inorganic porous scaffold may partially or completely become pore walls of the porous inverse carbon replica and the pore walls of the inorganic porous scaffold become the pores of the inverse carbon replica (Lu et al., 2004).

For the exemplary material described in this study, a task-specific PS, [1-(2-cyanoethyl)-2-phenylimidazolium][hydrogen sulfate] (Zhang et al., 2014b), and a silica-rich macroporous controlled porous glass (CPG) were applied as the starting materials for the process of nano-casting.

The specific PS was selected because of its (i) high nitrogen content, (ii) phenyl ring enabling easy graphitization on exposure to temperatures below 1000°C, and (iii) hydrogen sulfate ion enabling strong ionic bond integrity which, in turn, leads to increased carbon yield upon direct carbonization (Zhang et al., 2014b). The specific CPG was selected because of its following attractive properties (i) commercial availability, (ii) characteristically narrow pore size distribution, (iii) high-temperature resistance (up to 1200°C), and (iv) macroporosity (Inayat et al., 2013). The rationale for using a macroporous inorganic scaffold (CPG) was to allow a top-to-bottom approach wherein control over macropore (>50 nm pore size), mesopore, and micropore size distribution could be achieved by varying different parameters and materials during nano-casting.

CPG has been previously reported to be used for creating macroporous inverse carbon replica by Jian et al. (2008) with mesophase pitch as carbon precursor but the final material was free of dopants integrated in the bulk. In contrast, the findings of this study will demonstrate the application of nano-casting with CPG as a versatile means for creating well-defined nanoarchitectures following a top-to-bottom approach to control macro-, meso-, and microporosity. The use of PS as the carbon source during nano-casting will demonstrate the simultaneous control of the tuned/doped chemical character of the porous carbon replica. The successful removal of the porous inorganic scaffold using NaOH, an abundantly available and relatively less toxic reagent, will also be demonstrated. A controllable nanoarchitecture and presence of dopants are particularly desirable in potential applications such as gas separation, catalysis, and electrochemical devices.

## Materials and Methods

### Synthesis of Protic Salt

A defined amount of the weak base, 1-(2-cyanoethyl)-2-phenylimidazole (used as received from TCI chemicals), was added slowly to a stoichiometric quantity of 1 M aqueous solution of sulfuric acid. The mixture was stirred continuously for around 2 h (end-point: solution becomes clear). The excess water was removed using a rotary evaporator at 60°C and 60 mbar vacuum for 1 h (end-point: no further boiling observed). The formation of PS was confirmed by ^1^H and ^13^C nuclear magnetic resonance (NMR).

### Synthesis of Macroporous Carbon via Nano-casting

The synthesized PS was directly mixed with a macroporous glass (used as received from University of Leipzig Inayat et al., 2013 with a pore size of 100 nm (**Figure 3**), pore volume of 0.3 cm^3^/g (Table 1), particle size of 200–315 μm, and of granular shape) in a mass ratio of 2.5:1. Infiltration into the pores was ensured by increasing and maintaining the temperature of the mixture at 60°C for 3 h. The temperature was then raised at 0.5°C/min to 120°C and further increased to 600°C at 5°C/min at which it was maintained for 4 h in an inert atmosphere created by continuous flow of nitrogen. The composite was then added to a stoichiometrically excess quantity of 3 M aqueous NaOH solution for removing the silica-rich phase of the composite. The trapped air bubbles in the composite were evacuated with vacuum in a desiccator for 1 h. The solid composite was then ultrasonicated in NaOH solution for 15 min to break down the composite agglomerates thereby improving interfacial contact between NaOH and the composite. Finally, the composite was kept at 70°C for 3 days in the same NaOH solution under stirring. The recovered carbon replica (by filtration) was treated further with 0.2 M aqueous HCl solution overnight to neutralize any remaining NaOH. After the treatment with 0.2 M aqueous HCl, the carbon replica was washed thoroughly with distilled water and dried at 110°C overnight. Finally, the carbon replica was heat treated at 1000°C for 40 h upon ramping the temperature at 1°C/min from room temperature in nitrogen atmosphere to enhance its oxidation stability through graphitization.

**Table 1:** Derived values from N_2_ sorption isotherms at −195.8°C displayed in Figure 4.

**Material**	**BET surface area [m^**2**^/g]**	**Total pore volume at p/p_**0**_ ≈ 0.99 [cm^**3**^/g]**	**Micropore volume (α-s method) [cm^**3**^/g]**
Macroporous glass	25	0.3	0
Carbon replica	382	0.6	0.1
Heat-treated carbon replica^a^	75	0.5	0

a *Heat-treated at 1000°C for 40 h*.

### Characterization

The carbonaceous material prepared via the above procedure was characterized using the following techniques:

#### Scanning Electron Microscopy (SEM) Imaging

Zeiss Gemini Ultra 55 was used to record SEM images using the in-lens detector. The in-lens detector located along the same path on which the primary electron beam traverses captures secondary electrons of type 1 that are generated at or near the primary electron beam's impact point on the sample. Consequently, the in-lens detector provides direct information of the sample's surface. An acceleration voltage of 10 kV was used for the primary electron beam. A few milligrams of the sample were used for imaging without any pre-treatment such as sputtering.

#### Mercury Intrusion Porosimetry

Thermo Fisher Scientific Pascal 140 and Pascal 440 were used in conjunction to obtain the pore size distribution of the samples between 3.7 nm and 105 μm. Pascal 140 was used to prepare the sample by degassing in vacuum and performing low pressure measurements between 0.01 and 0.3 MPa. Pascal 440 was then used to perform high pressure measurements up to 400 MPa. Around 100–200 mg sample was used without any further pre-treatment.

#### N_2_ Physisorption Measurements

The sorption measurements were carried out using Quantachrome Quadrasorb SI at −195.8°C. Before the measurements, the samples were degassed with Quantachrome MasterPrep Degasser at 350°C under vacuum for 12 h. 35 to 85 mg of sample was used for the measurements. The advanced data treatment such as Density Functional Theory (DFT) based pore size distribution was carried out using the software Quantachrome QuadraWin version 5.05.

#### Thermogravitmetric (TG) Measurements

TG measurements were performed in TA Instruments TGA 2950. Five to ten milligram of sample was placed on platinum pan and exposed to a continuous flow of instrument air at a flow rate of 60 ml/min (STP). Temperature programmed oxidation was carried out with a ramp rate of 10°C/min from ambient temperature to 900°C. This instrument has a known accuracy of ±1% residual mass.

#### CHNS Elemental Analysis

CHNS analysis was performed in EuroVector EA 3000 by direct combustion of sample with oxygen. The flue gases were analyzed with a thermal conductivity detector to quantify the amount of carbon, hydrogen, nitrogen, and sulfur in terms of mass percent. A sample amount of 1–2 mg was used for the analyses. This instrument has an accuracy of ±0.5 mass % in the reported values.

#### Raman Spectroscopy

For a typical Raman measurement, a WiTec alpha300R confocal Raman microscope was used. A HeNe laser with an output of 633 nm was used for sample excitation. A typical spectrum was obtained for at least 5 different spots on the sample. Less than 5 mg of sample was used for the acquisition of the spectra.

Further characterization results from inductively coupled plasma—optical emission spectroscopy (ICP-OES) for CPG, NMR spectra for the synthesized PS, and X-ray diffraction for the heat-treated carbon replica can be found in the Supplementary Information.

## Results

### SEM-Imaging

**Figure 2:**
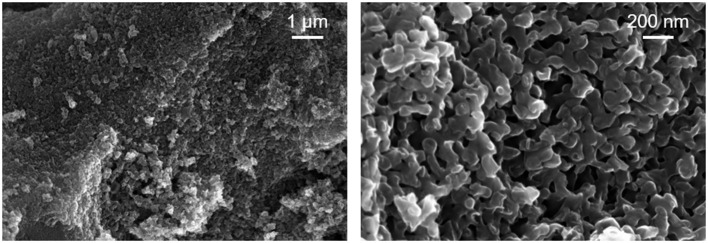
SEM images of the heat-treated carbon replica at two different magnifications.

The SEM images of the heat-treated macroporous carbon replica are displayed in Figure 2. The SEM image on the left-hand side of Figure 2 at lower magnification displays the textural homogeneity of the prepared material. In the right-hand side image of Figure 2, the expected pore wall thickness of around 100 nm can be readily observed along with macropores in the range of 50–75 nm. These results confirm that the 100 nm pores of the starting CPG used were transformed into pore walls of the carbon replica and the pore walls of the CPG were transformed into the pores of the carbon replica during the nanocasting process. Compared to reports of hierarchical N-doped carbon prepared with other methods (Eisenberg et al., 2016), the presence of macroporosity with high textural homogeneity was clearly demonstrated using SEM-imaging. The morphology of the heat-treated carbon replica was also found to be similar to inverse carbon replica reported previously in literature but with a larger pore diameter (Jian et al., 2008). The thick walls of the heat-treated macroporous carbon replica as seen in the SEM images may have had a positive impact on the thermal stability of the carbon replica.

### Mercury Intrusion Porosimetry

**Figure 3:**
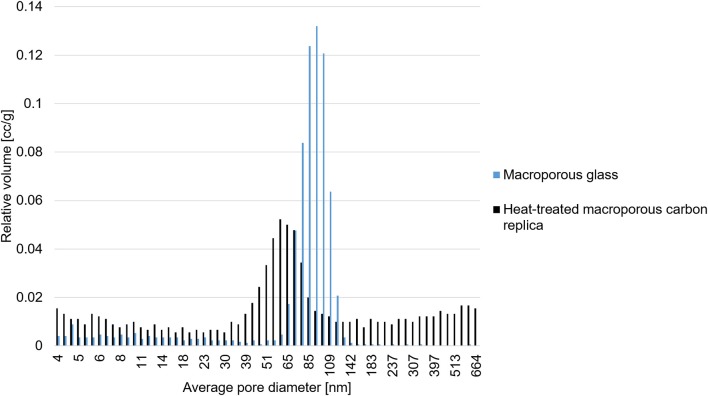
Estimated pore size distribution in the mesoporous and macroporous size range.

Figure 3 displays the meso- and macropore size distribution of the CPG and the heat-treated macroporous carbon replica which corroborates excellently with the findings from SEM-imaging. These findings also prove that the superficial pore size distribution apparent in the SEM images of Figure 2 is also found in the bulk of the material. The reported estimations were based on the assumption that the pores are cylindrical in shape and the contact angle of mercury with the material is 140°. The obtained results demonstrated the applicability of commercially available CPG to produce carbon replica with a well-defined nanoarchitecture. Typical macroporous carbon replica reported in literature have macropores in the size range of 200 nm (Sun et al., 2007; Zhong et al., 2009; Prévot et al., 2011) up to a micrometer (Cai et al., 2006). The reported material with its median pore width being approximately 60 nm was found to be rare in reported literature apart from the work by Eisenberg et al. (2016) which reported a median pore width slightly less than 50 nm for their N-doped hierarchical carbon.

### N_2_ Physisorption

**Figure 4:**
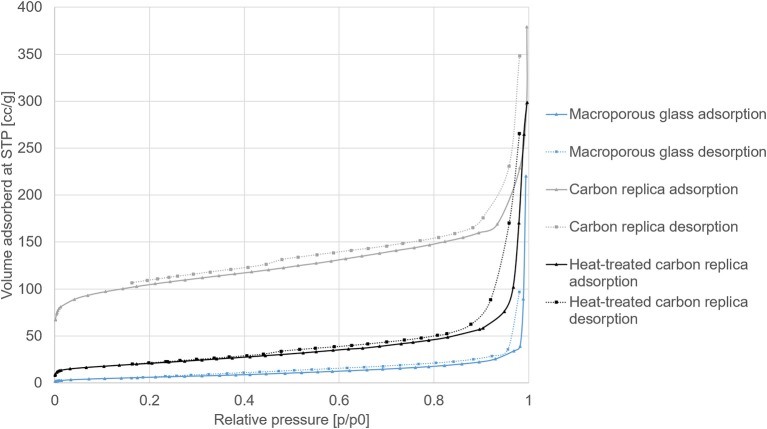
N_2_ sorption isotherms at −195.8°C of macroporous glass, its carbon replica, and the heat-treated carbon replica.

The N_2_ sorption isotherms of macroporous glass, its carbon replica, and the heat-treated carbon replica are displayed in Figure 4.

The steep rises at high p/p_0_ (>0.9) for all adsorption isotherms displayed in Figure 4 confirmed the presence of macropores. The hysteresis loops in the carbon replicas (gray and black isotherms) were indicative of the presence of additional mesopores. The derived values from the isotherms are displayed in Table 1. The relatively high BET surface area of the carbon replica before heat-treatment may be attributed to its microporosity as estimated by the α-s method for micropore analysis (selected points between p/p_0_ of 0.4 and 0.65). The microporosity appeared to have been generated by “activation” of the carbon by aqueous NaOH treatment during the etching step as the microporosity was not present in the carbonized composite before etching (not shown). This “activation” was similar to previously reported effects achieved by KOH (Sevilla and Fuertes, 2011; Hao et al., 2015b). This hypothesis was further corroborated by the fact that the PS used in this study, [1-(2-cyanoethyl)-2-phenylimidazolium][hydrogen sulfate], was previously reported to yield a non-microporous carbonaceous material upon standalone direct carbonization (Zhang et al., 2014a).

**Figure 5:**
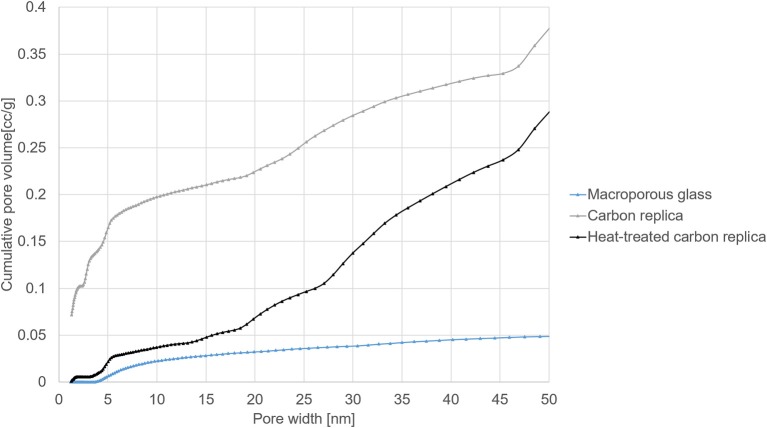
Pore size distributions in the micro- and mesoporous range from DFT modeling of N_2_ sorption isotherms at −195.8°C.

The pore size distributions displayed in Figure 5 were calculated by Density Functional Theory (DFT) modeling using the DFT kernel NLDFT (Non-Local Density Functional Theory) equilibrium model on silica assuming cylindrical pores for the macroporous glass. The kernel QSDFT (Quenched Surface Density Functional Theory) equilibrium model on carbon assuming cylindrical pores was used for calculating the pore size distribution of the carbon replica and the heat-treated carbon replica. The equilibrium model was selected for both types of materials to make use of the adsorption as well as desorption measurements for calculations. QSDFT model was used for porous carbon because it is known to predict pore size distributions of nanoporous carbon more accurately (Cychosz et al., 2017). Expected heterogeneity on the surface arising from hetero-atom doping and/or defect site also made QSDFT a more suitable choice.

DFT-modeled pore size distributions displayed in Figure 5 confirmed the loss of micropores after heat-treatment at 1000°C for 40 h. The loss of micropores and some small mesopores (size less than 3.5 nm) explains the reduction in total pore volume and the smaller hysteresis loop observed in heat-treated carbon replica. It should be noted that the micropore volume of about 0.1 cm^3^/g of the carbon replica estimated by DFT (displayed by the gray plot in Figure 5) corroborated very well with the micropore volume estimated by α-s method that is recommended by IUPAC (Thommes et al., 2015).

### TG Measurements in Air

**Figure 6:**
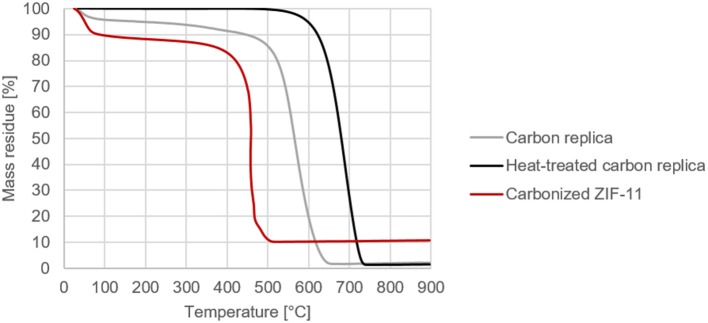
Comparative decomposition curves of carbon replicas in air (programmed temperature ramp from ambient conditions to 900°C at 10°C per min in a TG setup).

The beneficial effect of a further heat-treatment of the carbon replica becomes clear in Figure 6 which displays the TG results of the carbon replicas in air. An oxidation stability of up to 530°C in air was achieved without the use of a metal catalyst to enable graphitization (ICP-results confirming lack of metals in Supplementary Information) or the use of very high temperature in excess of 2000°C (Karthik et al., 2015). Apart from the much higher oxidation stability of the heat-treated carbon replica seen in Figure 5, its negligible mass loss at temperatures below 350°C also confirms the lack of micropores wherein water tends to be adsorbed at ambient conditions. For comparison purposes, ZIF-11 prepared based on the synthesis method by He et al. (2013) was carbonized at 1000°C (based on (Hao et al., 2015a) without activation using KOH) and tested under similar conditions. Compared to the carbonized ZIF-11, the heat-treated carbon replica and the carbon replica prior to heat-treatment showed significantly superior thermal stability and oxidation resistance.

### CHNS-Analysis

As expected, the CHNS results displayed in Table 2 confirmed the loss of nitrogen after the carbon replica was heat-treated. Nevertheless, a high nitrogen content of about 6 mass % compared to other N-doped porous carbon reported in literature (1.5–10 mass %, To et al., 2017) was retained in the carbon replicas. It is noteworthy that the hierarchically porous carbon replica before heat treatment possessed a very high nitrogen content of about 15 mass %. Moreover, our results (Figure 6 and Table 2) demonstrated the highly efficient removal of inorganic content with relatively benign aqueous NaOH instead of highly toxic HF which has been frequently used by other researchers during nano-casting with ionic liquids (Paraknowitsch et al., 2010b; Tzialla et al., 2016).

**Table 2:** CHNS-elemental analyses of carbon replicas.

**Sample**	**C**	**H**	**N**	**S**
Carbon replica^a^	70.7	2.6	15.0	0.4
Carbon replica after heat treatment	93.9	0.1	6.1	0.5

a *The remaining mass of carbon replica can be attributed to oxygen from desorbed water which corresponds with the estimated H values and typical mass loss at temperatures below 350°C in Figure 6 that can be expected from evaporation of water from the micropores*.

### Raman Spectroscopy

The G, D1, D3, and D4 Raman bands of the carbon replicas before and after heat-treatment were fitted in the ranges specified by Coccato et al. (2015) and Zhang et al. (2015c). D5-band positioned between 1,599 and 1,635 cm^−1^ was not fitted because of its virtual indistinguishability with G-band.

All the peaks were fitted with a 3-parameter Lorentzian function as recommended by Sadezky et al. (2005) on MATLAB^®^. The disordered to graphitic (D/G) ratio was calculated by dividing the sum of areas of D1, D3, and D4 peak by the area of the G peak.

**Figure 7:**
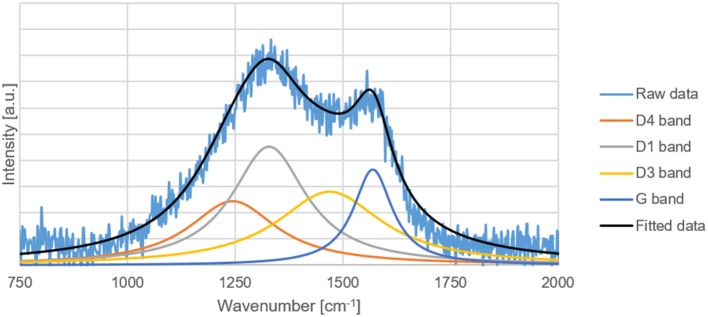
Raw and fitted Raman spectral data of the carbon replica.

**Figure 8:**
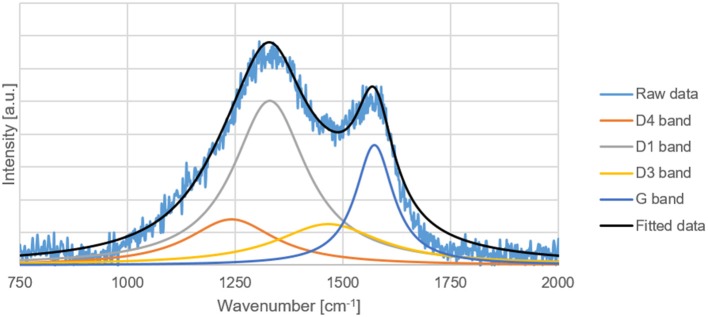
Raw and fitted Raman spectral data of the heat-treated carbon replica.

The D/G ratio of the carbon-replica upon heat-treatment decreases from 15.6 to 11.4 proving that graphitization occurs upon heat-treatment at a temperature of 1000°C. The increase in the intensity of G-peak can also be clearly observed in Figures 7, 8. These results are purely qualitative and a comparison with literature is ambiguous because of scarcity of publications distinguishing between the different bands that could possibly exist simultaneously apart from the D1 and G bands.

## Discussion

The application of nano-casting has the key advantage of allowing a methodical and precise approach for constructing the nano-architecture and tuning the chemical character of the inverse replica.

By the use of a macroporous CPG in this work, the feasibility of a top-to-bottom approach from a macroporous to microprous range was demonstrated. Before the heat treatment of the inverse carbon replica, a hierarchical carbonaceous material with micro-, meso-, and macroporosity was created, as proved by the N_2_ sorption results depicted in Figure 4. Such a material with high surface area and high nitrogen content could find application in CO_2_ capture (D'Alessandro et al., 2010; Zhang et al., 2015c).

It was demonstrated with Raman spectroscopy (Figures 7, 8) that a heat treatment could enhance the graphitization of the carbon replica and increase its oxidation stability as proved by TG results (Figure 6). A material with high oxidation stability and nitrogen doping is desirable for catalytic applications (Li et al., 2016). The combination of high oxidation stability, macroporosity, graphitic character for high electrical conductivity, and nitrogen doping is also suitable for electrocatalytic applications such as a catalyst support material for the oxygen reduction reaction (ORR) in a polymer electrolyte fuel cell (PEMFC). The ORR can be catalyzed by the nitrogen functionalities in the support material (Guo et al., 2016), the high oxidation stability is necessary to prevent oxidative corrosion that is common in the harsh chemical environment of a PEMFC (Reiser et al., 2005), and the macroporosity is desirable to avoid transport limitations in a PEMFC (Ous and Arcoumanis, 2013).

It was proved that a treatment of the composite material (carbon with the inorganic porous scaffold) with NaOH led to microporosity. Thus, for electrochemical applications such as supercapacitors which require high nitrogen content with micro-/mesoporosity and graphitic character (Wan et al., 2014), the removal of the inorganic porous scaffold can be carried out after the heat-treatment step.

Using a task-specific PS, the nitrogen doping of the inverse replica was implemented and a nitrogen content as high as 15 mass % (as proven by CHNS analysis in Table 2) was achieved. The nitrogen content was amenable to further tuning by an additional heat treatment with higher temperatures leading to reduced nitrogen content (Pels et al., 1995).

## Conclusion and Outlook

A well-defined macroporous carbon (50–100 nm pore width) with high nitrogen content (6–15 mass. %) and oxidation stability up to 530°C was prepared using a novel, easy-to-synthesize carbon source and commercially available porous glass via nano-casting. By characterizing the material at different stages of nano-casting, the flexibility of nano-casting was demonstrated and the evolution of the material properties during the different stages of the process was revealed. The approach could be extended to create a variety of doped porous carbons with defined nanoarchitecture by matching a suitable PS with an appropriate CPG with the desired pore size distribution. Materials tailored for advanced applications including but not limited to gas separation, catalysis, and electrochemical devices could be synthesized based on the approach outlined here. It is thus of interest to further apply the approach to create and test materials developed with this approach for their end-applications. The use of environmentally benign materials for the preparation of doped, porous carbon makes it a very promising approach to be studied for possible scale-up of the synthesized materials.

## Data Availability Statement

All datasets generated for this study are included in the article/Supplementary Material.

## Author Contributions

VS synthesized the carbon and characterized it. VS and MG prepared the protic salt together. MG performed the NMR. MM and DE provided the CPG. TS and WS directed the work and assisted in experimental design. All authors discussed the data and provided feedback.

### Conflict of Interest

The authors declare that this study received funding from Dyneon GmbH. Dyneon GmbH was not involved in the study design, collection, analysis, interpretation of data, the writing of this article or the decision to submit it for publication.
