# Supplementary material for: Nano-casted N-Doped Carbon Created From a Task-Specific Protic Salt and Controlled Porous Glass
Source: Front Chem. 2019 Nov 27;7:767. doi: 10.3389/fchem.2019.00767 (PMC6897309; doi:10.3389/fchem.2019.00767)
Supplement: Supplementary file 1 [file Data_Sheet_1.docx]

Supplementary Material for nano-casted N-doped carbon created from a task-specific protic salt and controlled porous glass

Varun Singh^1,†^, Mikhail Gantman^1^, Thangaraj Selvam^1^, Maximilian Münzer^2^, Dirk Enke^2^, and Wilhelm Schwieger^1*^,

^1^Institute of Chemical Reaction Engineering, Friedrich-Alexander University of Erlangen-Nuremberg (FAU), Egerlandstrasse 3, 91058 Erlangen, Germany.

^2^Institute of Chemical Technology, Universität Leipzig, Linnéstrasse 3, 04103 Leipzig, Germany.

^†^Current address: Laboratory for Chemical Technology, Ghent University, Technologiepark 125, B-9052 Ghent, Belgium.

*** Correspondence:**Wilhelm Schwieger
wilhelm.schwieger@fau.de

Keywords: N-doped carbon, macroporous carbon replica, direct carbonization, controlled porous glass, nano-casting.

- Inductively coupled plasma - optical emission spectroscopy (ICP-OES) based investigation of macroporous glass and its heat-treated carbon replica for trace metals.
- ^1^H and ^13^C NMR of 1-(2-cyanoethyl)-2-phenylimidazole and protic salt, [1-(2-cyanoethyl)-2-phenylimidazolium][hydrogen sulfate].
- X-ray diffraction.

# ICP-OES investigation to find trace metal elements

No traces of metals apart from sodium (1.7 wt.%) and aluminum (0.029 wt.%) were found in the heat-treated carbon replica. Sodium may have been detected because the carbon replica was in contact with 3 M aqueous NaOH prior to its heat treatment. Traces of aluminum may be attributed to the remains of the macroporous glass used during the process of preparation of the carbon replica. The digestion mixture used for the analysis contained 6 ml 37% HCl, 4 ml 65% HNO_3_, and 2 ml 40% HF. The sample amount for the analysis was 53 mg and the sample dissolved in the digestion mixture was further treated with microwaves to enhance complete leaching of trace elements.

For the ICP-OES analysis of macroporous glass, very small traces of iron were detected (0.0039 wt.%) and, as expected, aluminum (0.113 wt.%). Prior to the analysis, a sample amount of 84 mg was dissolved in a digestion mixture containing 3 ml 37% HCl, 2 ml 65% HNO_3_, and 8 ml 40% HF and thereafter, exposed to microwaves. The extremely small amount of iron detected in the bulk macroporous glass is unlikely to have played a major catalytic role in the graphitization of the carbon replica. This is further corroborated by the fact that iron was not detected in the heat-treated carbon replica. The lack of any iron in the heat-treated carbon replica may be attributed to the loss of even minute quantities of iron during the overnight acid treatment of the carbon replica (before the heat-treatment) with 0.2 M HCl aqueous solution prior to its heat-treatment. These results thus prove that graphitization and increased oxidation stability can be attributed to the chemical structure of the protic salt.

# ^1^H and ^13^C NMR in Tridueterio(trideuteriomethylsulphinyl)methane [DMSO-d_6_]

Comparison of the ^1^H spectra of 1-(2-cyanoethyl)-2-phenylimidazole and [1-(2-cyanoethyl)-2-phenylimidazolium][hydrogen sulfate] in Figure S-1 and Figure S-3, respectively along with ^13^C spectra of 1-(2-cyanoethyl)-2-phenylimidazole and [1-(2-cyanoethyl)-2-phenylimidazolium][hydrogen sulfate] in Figure S-2 and Figure S-4, respectively confirm the formation of the protic salt. The NMR spectra of the protic salt correspond well with those reported previously in literature (see supporting information of (Zhang, et al. 2014)). The broad peak between 5 and 6 ppm in Figure S-3 may be explained by the presence of residual water in [1-(2-cyanoethyl)-2-phenylimidazolium][hydrogen sulfate]. Using Karl-Fischer titration, the amount of water in the protic salt at the time of measurement was estimated to be about 3.6 wt. %.


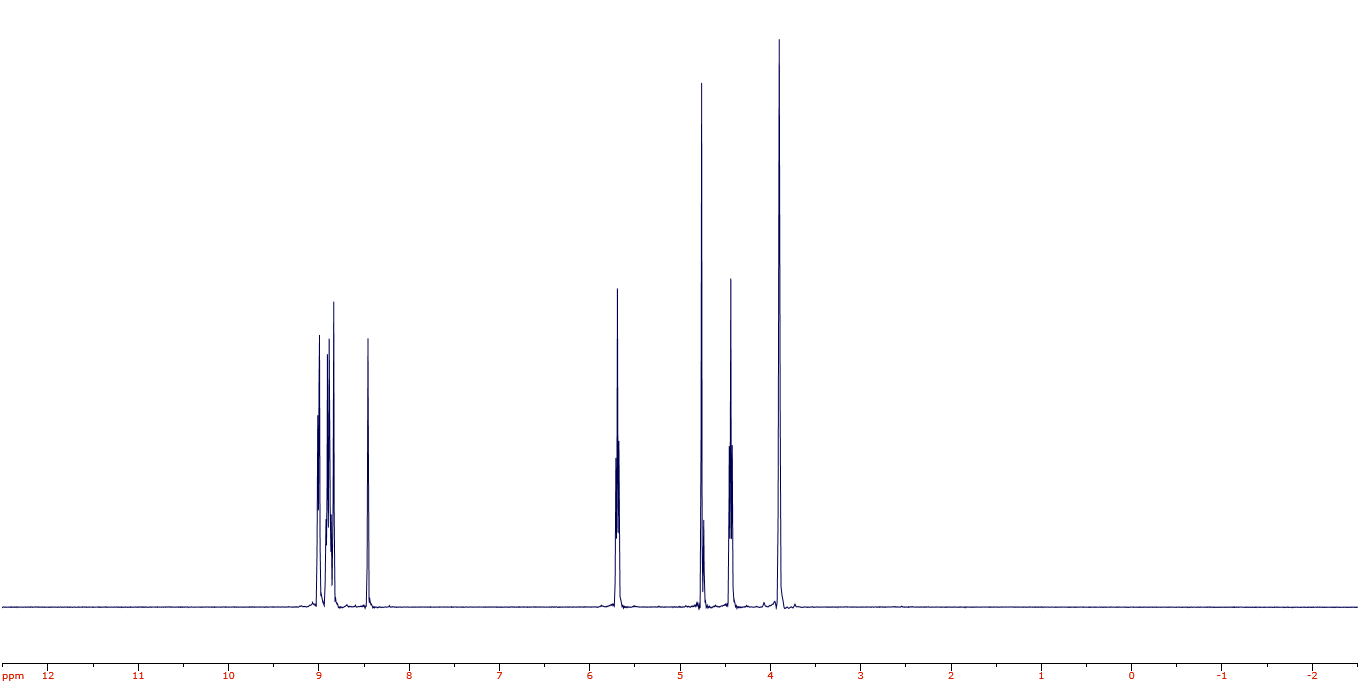


Figure S-1 ^1^H NMR spectra of 1-(2-cyanoethyl)-2-phenylimidazole. In the plot, x-axis represents the chemical shifts in ppm and y-axis the peak intensities.


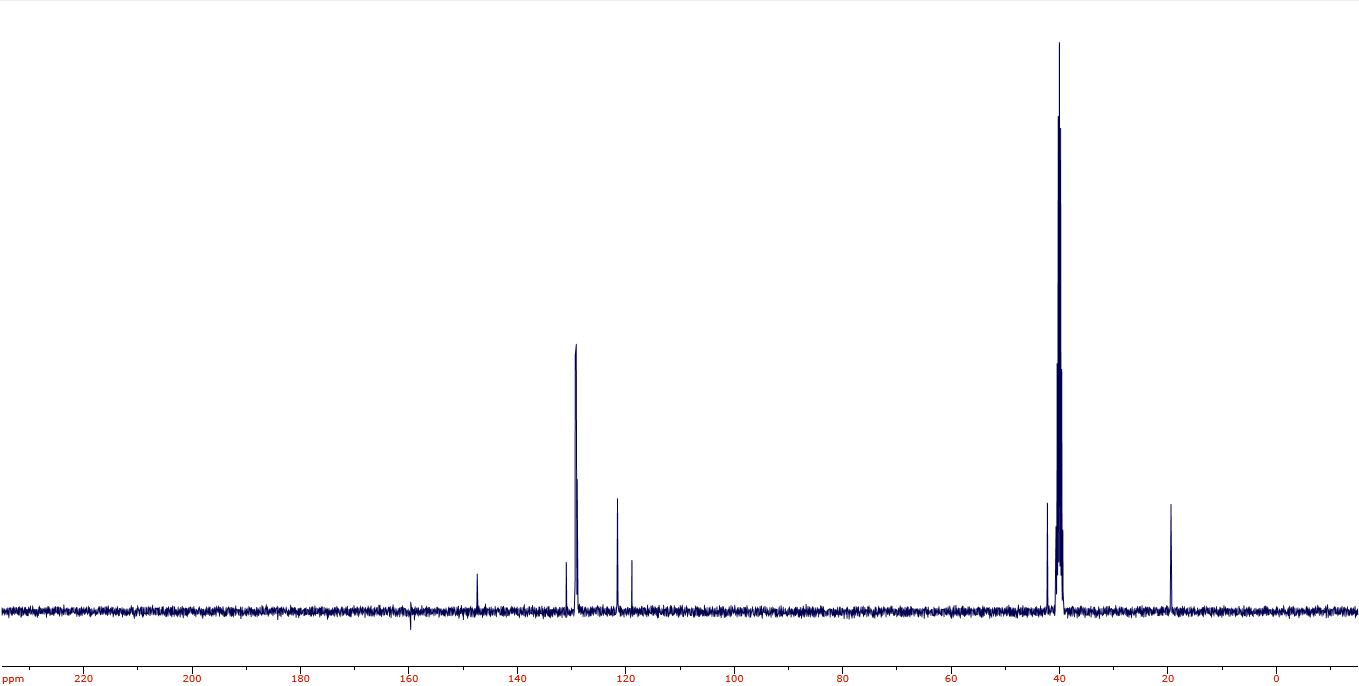


Figure S-2 ^13^C NMR spectra of 1-(2-cyanoethyl)-2-phenylimidazole. In the plot, x-axis represents the chemical shifts in ppm and y-axis the peak intensities.


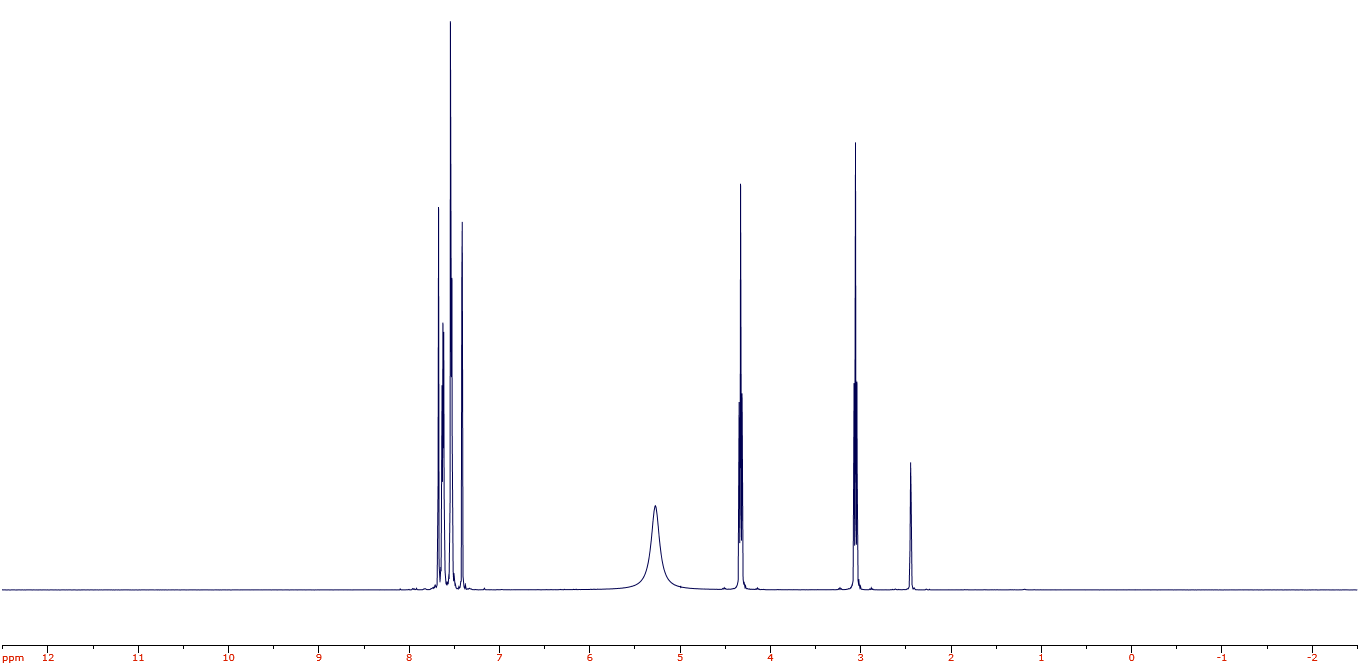


Figure S-3 ^1^H NMR spectra of [1-(2-cyanoethyl)-2-phenylimidazolium] [hydrogen sulfate]. In the plot, x-axis represents the chemical shifts in ppm and y-axis the peak intensities.


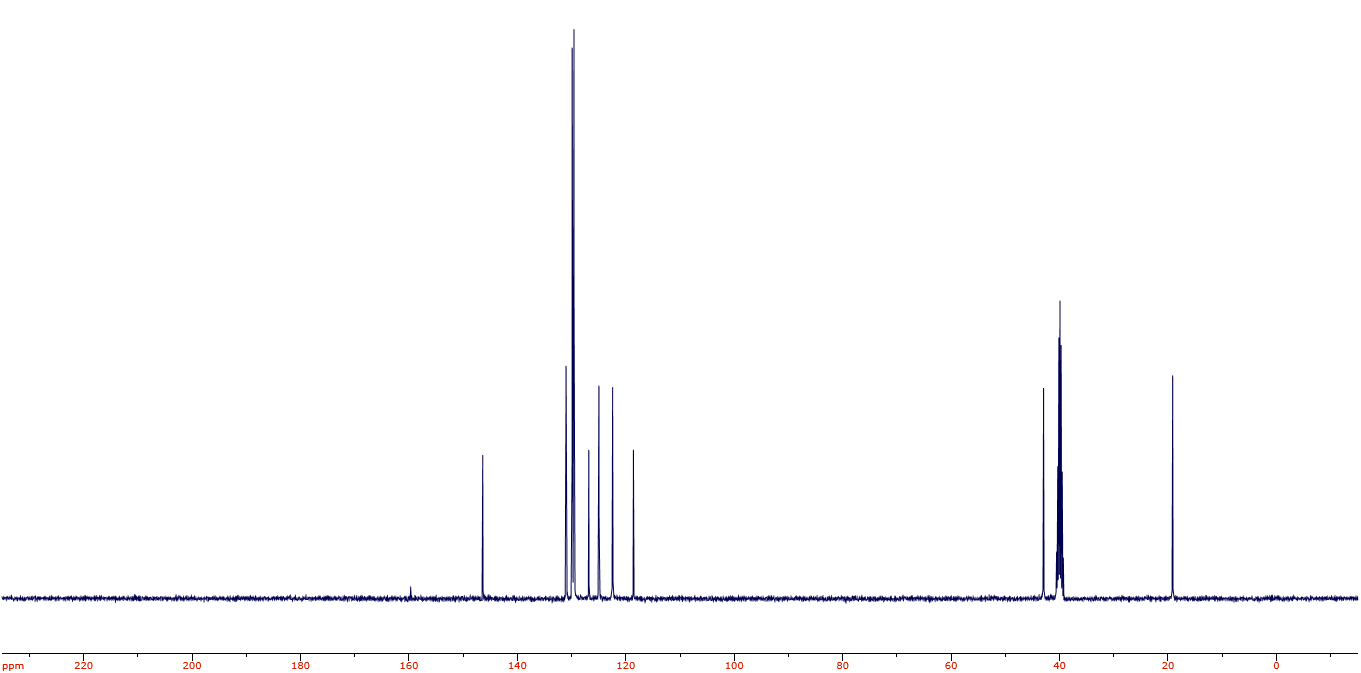


Figure S-4 ^13^C NMR spectra of [1-(2-cyanoethyl)-2-phenylimidazolium][hydrogen sulfate]. In the plot, x-axis represents the chemical shifts in ppm and y-axis the peak intensities.

# X-ray diffraction

Philips X’pert multi-purpose diffractometer (MPD) DY1202 with Cu-Kα (α-1 and α-2) as radiation source (λ = 1.54056 Å) was used to obtain the X-ray diffractograms. The defined measurement range was 2 - 50° (2θ) and the capture rate was 0.013°/second.

Figure S-5 displays the X-ray diffractogram of the heat-treated carbon replica and a comparison of it to a commercially available graphitised carbon black (Ketjenblack EC-300JD graphitised by Tanaka Kikinzoku Kogyo K.K.). The (002) peak at 25.8° and (100) peak at 41° indicating a graphitic character are clearly visible in both materials whereas the characteristic (101) graphitic peak expected between 44 and 45° is not distinct in either samples. The good match between the peaks of the heat-treated carbon replica and a commercially available graphitized (un-doped) carbon black further corroborates to the hypothesis that the heat-treated carbon replica possesses a graphitic character.

*
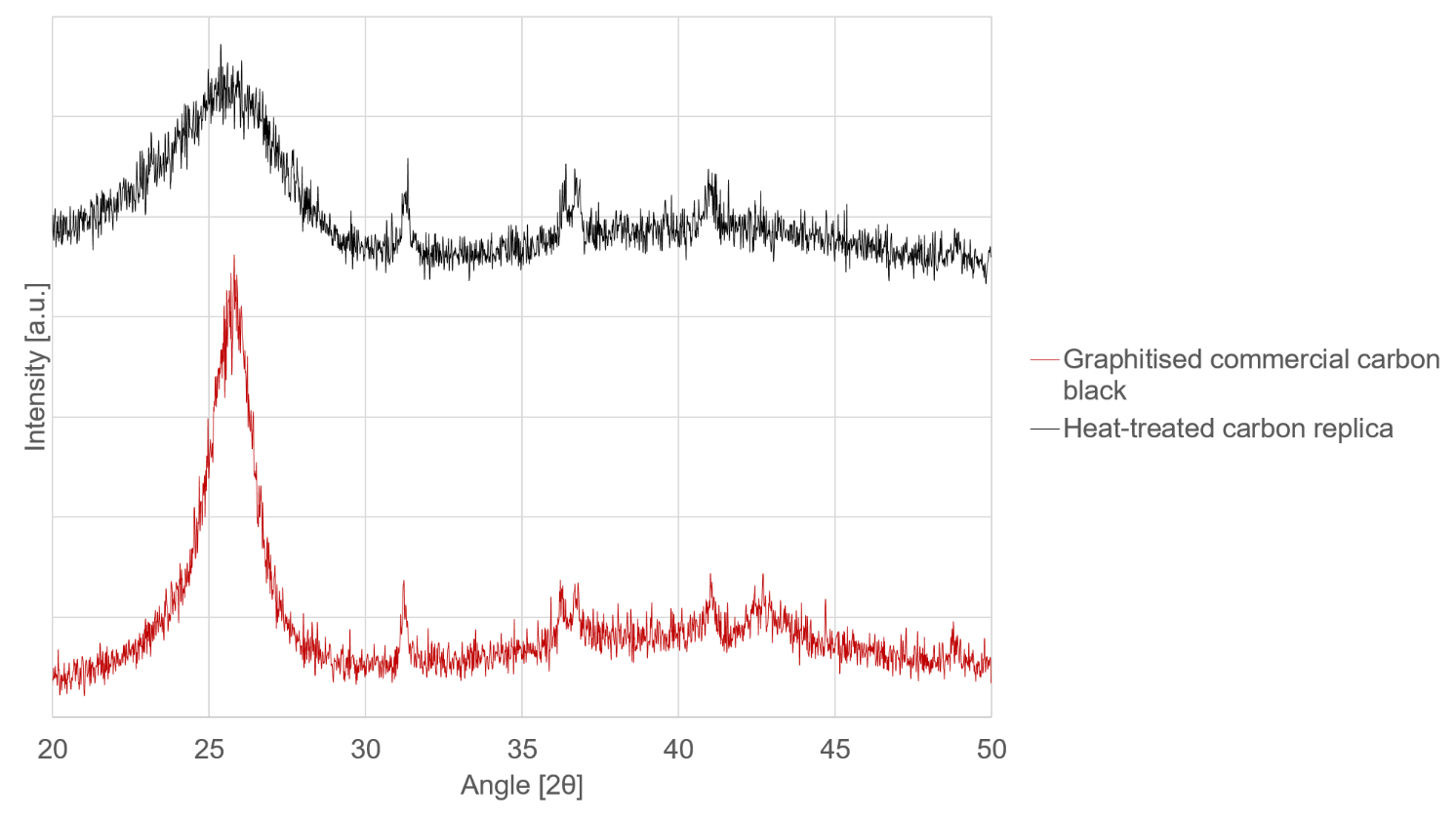
*

Figure S-5: X-ray diffractograms of the heat-treated carbon replica and a typical commercial graphitized carbon black.

# References

Zhang, Shiguo, Muhammed Shah Miran, Ai Ikoma, Kaoru Dokko, and Masayoshi Watanabe. 2014. "Protic ionic liquids and salts as versatile carbon precursors." *Journal of the American Chemical Society* (American Chemical Society) 136: 1690-1693. doi:10.1021/ja411981c.
